# Efficacy and safety of remote ischemic conditioning for acute ischemic stroke: A comprehensive meta‐analysis from randomized controlled trials

**DOI:** 10.1111/cns.14240

**Published:** 2023-05-14

**Authors:** Xiuji Kan, Zeya Yan, Fei Wang, Xinyu Tao, Tao Xue, Zhouqing Chen, Zhong Wang, Gang Chen

**Affiliations:** ^1^ Department of Neurosurgery The First Affiliated Hospital of Soochow University Suzhou China; ^2^ Suzhou Medical College of Soochow University Suzhou China; ^3^ Department of Neurosurgery & Brain and Nerve Research Laboratory The First Affiliated Hospital of Soochow University Suzhou China; ^4^ Department of Neurosurgery, Beijing Tiantan Hospital Capital Medical University Beijing China

**Keywords:** ischemia, meta‐analysis, randomized controlled trials, remote ischemic conditioning, stroke

## Abstract

**Background and Purpose:**

Remote ischemic conditioning (RIC) is a remote, transient, and noninvasive procedure providing temporary ischemia and reperfusion. However, there is no comprehensive literature investigating the efficacy and safety of RIC for the treatment of acute ischemic stroke. In the present study, we performed a comprehensive meta‐analysis of the available studies.

**Methods:**

MEDLINE, Embase, the Cochrane Library database (CENTRAL), and ClinicalTrials.gov were searched before Sep 7, 2022. The data were analyzed using Review Manager 5.4.1 software, Stata version 16.0 software, and R 4.2.0 software. Odds ratio (OR), mean difference (MD), and corresponding 95% CIs were pooled using fixed‐effects meta‐analysis.

**Results:**

We pooled 6392 patients from 17 randomized controlled trials. Chronic RIC could reduce the recurrence of ischemic stroke at the endpoints (OR 0.67, 95% CI [0.51, 0.87]). RIC could also improve the prognosis of patients at 90 days as assessed by mRS score (mRS 0–1: OR 1.29, 95% CI [1.09, 1.52]; mRS 0–2: OR 1.22, 95% CI [1.01, 1.48]) and at the endpoints assessed by NIHSS score (MD −0.99, 95% CI [−1.45, −0.53]). RIC would not cause additional adverse events such as death (*p* = 0.72), intracerebral hemorrhage events (*p* = 0.69), pneumonia (*p* = 0.75), and TIA (*p* = 0.24) but would inevitably cause RIC‐related adverse events (OR 26.79, 95% CI [12.08, 59.38]).

**Conclusions:**

RIC could reduce the stroke recurrence and improve patients' prognosis. Intervention on bilateral upper limbs, 5 cycles, and a length of 50 min in each intervention might be an optimal protocol for RIC at present. RIC could be an effective therapy for patients not eligible for reperfusion therapy. RIC would not cause other adverse events except for relatively benign RIC‐related adverse events.

## Graphical abstract

This meta‐analysis pooled 6392 patients from 17 randomized controlled trials. The main findings were as follows: RIC could reduce the recurrence of stroke and improve patients' prognosis. Intervention on bilateral upper limbs, 5 cycles, and a length of 50 min in each intervention might be the optimal protocol for RIC at present. RIC could be an effective therapy for patients not eligible for reperfusion therapy. RIC would not cause other adverse events except for relatively benign RIC‐related adverse events.

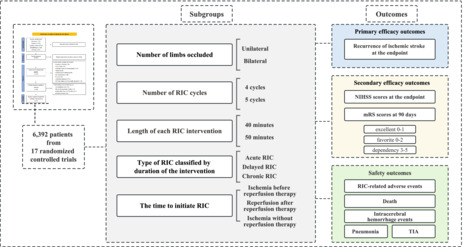

## 1 INTRODUCTION

Stroke is the second leading cause of mortality and disability worldwide, accounting for 6.55 million deaths and 143 million disability‐adjusted life years per year.^1^ The most common type of stroke is the ischemic stroke (IS), which accounts for 87% of all strokes.^2^ For these patients, the most intuitive means of treatment to restore blood flow before major brain damage occurs is to remove the blockage by a combined injection of clot‐dissolving drugs or use of a mechanical device, or both.^3^, ^4^, ^5^ Unfortunately, only less than 5% of stroke patients can benefit from these therapies, mainly due to the narrow therapeutic window and the risk of inducing ischemia–reperfusion injury (IRI), increasing lesion size and worsening blood barrier breakdown leading to cerebral edema and hemorrhage.^6^, ^7^, ^8^, ^9^, ^10^

In view of this condition, an innovative and effective way to extend the therapeutic windows and to mitigate further brain injury is urgently needed. By targeting multiple molecular pathways associated with cell death, remote ischemic conditioning (RIC) is thought to remotely recruit neuroprotective pathways as a neuroprotective strategy.^11^ RIC is an inexpensive, remote, noninvasive intervention that has been successfully demonstrated in several preclinical studies in the kidney, heart, and brain since its inception in 1986.^11^, ^12^, ^13^ RIC provides temporary episodes of reversible ischemia through repeated inflations and deflations of a blood pressure limb cuff, with the intent of protecting remote organs such as the brain or heart from subsequent ischemic injury.^14^ RIC can be divided into three types based on the initiation of conditioning: preconditioning (RIPreC, RIC applied before ischemia), perconditioning (RIPerC, RIC applied after the onset of ischemia and before reperfusion), and postconditioning (RIPostC, RIC applied during reperfusion).^15^ Currently, the possible mechanisms of RIC include neurovascular protection by inducing anti‐inflammatory effects and neuronal protection against excitotoxicity, coupled with mitochondrial protection, circulating inflammasome activation and/or transcriptional regulation of neuroprotective pathways, and enhancement of collateral circulation.^16^, ^17^, ^18^, ^19^

Although there have been previous systematic reviews and meta‐analyses of RIC as a neuroprotective therapy, most of them focused on preclinical animal models.^20^, ^21^, ^22^, ^23^, ^24^ However, translating treatment effects on stroke from animal ischemia models to clinical reality is often a great challenge due to nonphysiological experimental conditions in animals that do not reflect human conditions.^25^ In addition, others made the summary of the completed RCTs on RIC in IS patients with a high percentage of participants lost to follow‐up, a limited number of recruited subjects, and a lack of comprehensive analyses.^26^, ^27^, ^28^, ^29^ The standard for the optimal protocol and initiation time of RIC is important but still inconclusive.^30^

Given that a systematic evaluation of the efficacy and safety of RIC in AIS and the optimal protocol of RIC has not yet been performed, we conducted a study to provide comprehensive evidence of the neuroprotective effects of RIC on patients with AIS through a meta‐analysis.

## 2 METHODS

A meta‐analysis was performed in conformity with the Preferred Reporting Items for Systematic Reviews and Meta‐Analyses (PRISMA, Table S1) guidelines.^31^ We did not prepare a protocol and our study was not registered.

### 2.1 Search strategy

We systematically searched MEDLINE, Embase, the Cochrane Library database (CENTRAL), and ClinicalTrials.gov for any peer‐reviewed research articles published before Sep 7, 2022. Search keywords included two aspects, including stroke and RIC, and search terms were adjusted for different databases. After removing duplicate and not directly relevant studies, two investigators (XJK and FW) screened each remaining article by reading the title, abstract, etc., to determine whether the research met the predefined inclusion criteria. Disagreements between the two investigators were resolved through sufficient discussion. In case of persistent divarication, a third investigator (ZYY) intervened to resolve the divarication. Detailed search strategies are provided in Table S2.

### 2.2 Inclusion/Exclusion criterion

Studies were included in our research if (a) the type of studies were randomized controlled trials (RCTs); (b) participants were adults with the onset of acute ischemic stroke based on a combination of clinical examination results, according to the diagnostic criteria for AIS used by researchers to enroll patients in their RCTs; (c) the RIC intervention consisted of RIPreC, RIPerC, and RIPostC, which were the same procedure but differed based on the protocol; (d) the control consisted of the sham group that required application of a blood pressure cuff or other occlusive devices (without complete blood flow occlusion such as cuff inflation to 30 mmHg) or blank group without sham procedure.

Studies were excluded if (a) the type of study was a single‐arm trial, completed RCTs but without available results and RCTs with low compliance rate and non‐RCTs; (b) participants were diagnosed with gradual‐onset cerebral ischemia such as cerebral small‐vessel disease and patients undergoing cardiovascular interventional surgery or any other type of cardiac surgery; (c) studies without outcomes of the present research.

### 2.3 Outcome measures

The primary efficacy outcome was the recurrence of ischemic stroke at the endpoint. The secondary efficacy outcomes were based on the National Institutes of Health Stroke Scale (NIHSS, range: 0–42, with higher scores indicating more severe neurological impairment) scores, which is the most widely used measure of the severity of presenting stroke and a dominant predictor of patient functional outcome,^32^ and modified Rankin Scale (mRS, range: 0–6, with higher scores indicating worse prognosis) scores, which is considered to be the standard clinical endpoints in acute stroke trials.^33^ The secondary efficacy outcomes included the following: NIHSS score at the endpoint, excellent outcome (mRS 0–1) at 90 days, favorite outcome (mRS 0–2) at 90 days, and dependency (mRS 3–5) at 90 days. The safety outcomes were RIC‐related adverse events including arm pain assessed by visual scale, redness or swelling of arms, skin petechiae on arms, dizziness or nausea and headache, death, intracerebral hemorrhage events including intracerebral hemorrhage and hemorrhagic transformation, pneumonia and TIA.

### 2.4 Data extraction

Two investigators (XJK and FW) independently extracted data from each included study and recorded the details on an extraction form. The extracted data consisted of three aspects as follows: (a) baseline information of the included trials (first author, year, registration number, publication, country, total number of participants, type of disease), patient characteristics (age range, mean age, gender, details of receiving reperfusion therapy, NIHSS score), and outcome events; (b) parameters of RIC: type of RIC classified by the time to initiate the RIC (RIPreC, RIPerC, or RIPostC), number of limbs occluded (unilateral or bilateral), number of RIC cycles, length of each RIC intervention, duration of the overall intervention and the time to initiate RIC; (c) data related to outcome measures: time of measurement (e.g., mRS score at 90 days), the number of participants with the event in each group. All extracted data were thoroughly reviewed by the same two authors, and discrepancies were resolved by sufficient discussion with a third reviewer (ZYY). Where necessary, data were extracted from graphs using GetData Graph Digitizer software.

### 2.5 Data analysis

The study data were analyzed using Review Manager 5.4.1 software (RevMan 5.4.1), Stata Version 16.0 software, and R 4.2.0 software. For continuous data, mean differences (MD) and their corresponding 95% confidence intervals (95% CI) were calculated using the inverse variance method with a fixed‐effect model. For dichotomous data, odds ratios (ORs) and their corresponding 95% CIs were calculated with the Mantel–Haenszel method, also using a fixed‐effect model. Statistical heterogeneity between studies was assessed using Chi‐squared and *I* ^2^ statistics. The statistical test of heterogeneity was considered significant if the *p*‐value was <0.10, and we defined a value greater than 50% as substantial heterogeneity. Where there was substantial heterogeneity, a heterogeneity test was performed using a Labbe plot, followed by a sensitivity analysis was conducted. Publication bias was assessed using the Egger weighted regression statistic and visual inspection of contour‐enhanced funnel plots.^34^

### 2.6 Subgroup analysis

We performed subgroup analyses to examine heterogeneity by analyzing differences in the number of limbs occluded (unilateral or bilateral), the number of RIC cycles (4 or 5 cycles), the length of each RIC intervention (40 or 50 min), the type of RIC classified by the duration of the intervention (acute, delayed and chronic), and the time to initiate RIC (during ischemia before reperfusion therapy, during reperfusion after reperfusion therapy, during ischemia without reperfusion therapy). Worth mentioning, in the subgroup of type of RIC,^11^ acute RIC is performed during the transfer of patients to comprehensive stroke centers, for use with reperfusion therapy (e.g., 1 session of RIC/day within 24 h of onset); chronic RIC is performed daily for several months for chronic neurological conditions such as intracranial atherosclerotic stenosis (ICAS) (e.g., 1 session of RIC/day for no less than 180 days); and the duration of intervention of delayed RIC is between the first former two types (e.g., 1 session of RIC/day for more than 24 h to no more than two weeks). In addition, whether RIC was used as a primary or adjuvant therapy to reperfusion therapy was important, because the timing of therapy initiation has received considerable attention.^30^ In the present study, patients who received reperfusion therapy accepted RIC therapy during ischemia or reperfusion, and those who did not receive reperfusion therapy accepted RIC during different phases of ischemia.

### 2.7 Risk of bias and quality assessment

Two investigators (XJK and FW) independently assessed the risk of bias for each study using the criteria outlined in the Cochrane Handbook for Systematic Reviews of Interventions.^35^ Disagreements were resolved by discussion or, if necessary, by consultation with a third investigator (ZYY). We assessed the risk of bias according to the following domains: selection bias, performance bias, detection bias, attrition bias, reporting bias, and other possible bias. For each domain, we classified each stud as “low risk,” “unclear risk,” or “high risk.” We summarized the evidence by creating “Summary of findings” tables using GRADEproGDT (https://gradepro.org/), into which we imported data from RevMan 5.4.1. We used the GRADE approach to rate the quality of evidence as high, moderate, low, or very low, according to the following five considerations: study limitations, imprecision, consistency of effect, indirectness, and publication bias.

## 3 RESULTS

### 3.1 Description of included studies

**FIGURE 1:**
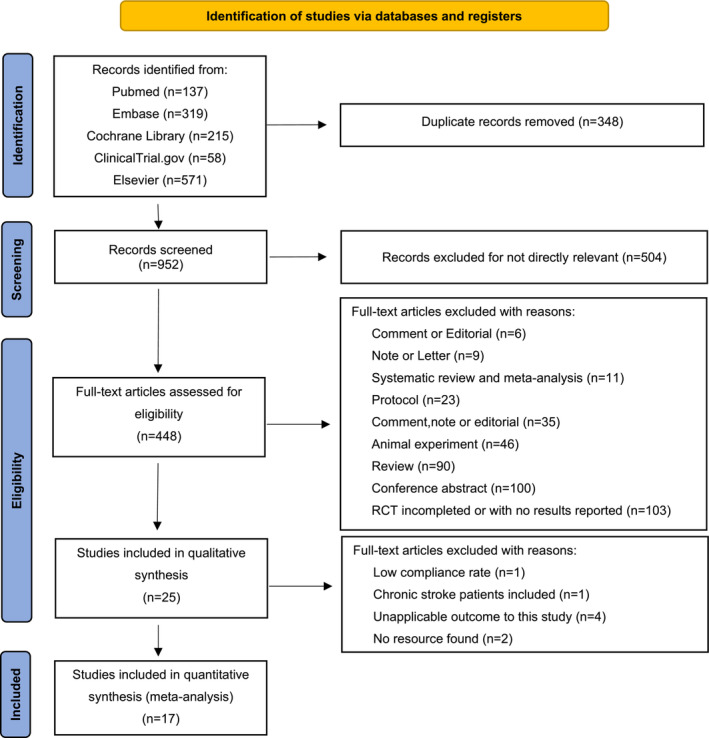
Preferred reporting items for systematic reviews and meta‐analysis (PRISMA) flow diagram.

The study selection process has been shown in the flow diagram (Figure 1). Using the search strategy described above, a total of 1299 records were retrieved from preselected databases by Sep 7, 2022. After removing duplicates and records that were not directly relevant, 17 studies remained and were pooled in meta‐analysis after applying the exclusion criteria. The primary information of the included studies is shown in Table 1. A total of 6392 patients were included in the analysis (3185 in the RIC group and 3207 in the control group), predominantly male (61.9%, 60.9% in the RIC group, and 63.0% in the control group) and Chinese (86.6%, 86.1% in the RIC group and 88.0% in the control group). Among the different studies, 4 included 3348 patients diagnosed with ICAS of at least 70% measures by ultrasound or other imageological examinations, who had an onset of AIS or TIA within 6 months^36^ (*n* = 1) or 7 days^37^ (*n* = 1) or 30 days^38^, ^39^ (*n* = 2) before enrollment. The remaining 13 trials enrolled 3044 participants with AIS whose time of RIC initiation from stroke onset was earlier, ranging from within 6 h to within 72 h. RIC protocols varied with the use of a blood pressure cuff, tourniquet, or cross‐clamp at a remote site (upper limbs or lower limbs), and protocol details are shown in Table S3.

**TABLE 1:** Summary of studies included in the meta‐analysis.

Study	Publication	Total number, Country	Type of disease	Age range	Mean age ± SD (year)	Male (*n*, %)	Treatment group, control group (No.of participants)	Reperfusion therapy (*n*, %)	NIHSS score (mean ± SD)	Outcome
An et al., 2020^40^ (NCT03218293)	Neurology	68, China	AIS	32 ~ 84	RIPC (62.06 ± 12.1) Blank (67.09 ± 9.9)	RIPC (22.64.7%) Blank (25.73.5%)	RIPC (*n* = 34) Blank (*n* = 34)	Received IVT with rt‐PA (68,100%)	RIPC (6.6 ± 1.7) Blank (5.3 ± 2.0)	c, d, e, g, h
Che et al., 2019^59^ (NCT03231384)	Annals of Clinical and Translational Neurology	30, China	AIS	≥18	RIPC (66.1 ± 11.2) Blank (65.3 ± 9.4)	RIPC (11,73.3%) Blank (13,86.7%)	RIPC (*n* = 15) Blank (*n* = 15)	Received IVT with rt‐PA (30,100%)	RIPC (7.3 ± 1.4) Blank (5.5 ± 1.7)	a, b, f, h, j
Chen et al., 2022^60^ (NCT03740971)	JAMA	1893, China	Acute moderate ischemic stroke	≥18	RIC (65.3 ± 10.5) Blank (65.3 ± 10.1)	RIC (556,64.4%) Blank (614,67.3%)	RIC (*n* = 922) Blank (*n* = 971)	Not received IVT	RIC (7.3 ± 0.5) Blank (7.3 ± 0.5)	a, c, d, e, f, g, h, i
England et al., 2017^61^ (ISRCTN86672015)	Stroke	26, United Kingdom	AIS	≥18	RIC (74.4 ± 10.8) Sham (77.7 ± 10.2)	RIC (8,61.5%) Sham (9,69.2%)	RIC (*n* = 13) Sham (*n* = 13)	Not received IVT	RIC (6.9 ± 2.5) Sham (5.8 ± 1.8)	a, b, g
England et al., 2019^62^ (NCT02779712)	Journal of the American Heart Association	60, United Kingdom	Hyperacute IS	≥18	RIC (70.9 ± 13.4) Sham (73.7 ± 10.2)	RIC (21,70%) Sham (15,50.0%)	RIC (*n* = 31) Sham (*n* = 29)	Received thrombolysis (33, 55.0%)	RIC (6.0 ± 1.5) Sham (7.3 ± 2.2)	a, b, c, d, e, g, h, i, j
He et al., 2020^53^ (NCT04027621)	Annals of Clinical and Translational Neurology	49, China	AIS	18 ~ 80	RIC (59.5 ± 8.5) Sham (61.3 ± 11.0)	RIC (20,83.3%) Sham (18,72.0%)	RIC (*n* = 24) Sham (*n* = 25)	Received IVT with rt‐PA (49, 100%)	RIC (7.8 ± 1.3) Sham (8.8 ± 1.5)	a, b, c, d, e, g, h, i
Hou et al., 2022^55^ (NCT02534545)	Lancet Neurology	3033, China	IAS with onset of AIS or TIA	40 ~ 80	RIC (61.1 ± 9.1) Sham (61.0 ± 9.1)	RIC (981, 64.7%) Sham (973, 64.2%)	RIC (*n* = 1517) Sham (*n* = 1516)	Not received IVT	/	a, f, g, h, j
Hougaard et al., 2013^54^ (NCT00975962)	Stroke	453, Denmark	AIS	≥18	rPerC (66.5 ± 3.4) Blank (67.8 ± 3.3)	rPerC (91,57%) Blank (74,59%)	rPerC (*n* = 247) Blank (*n* = 196)	Received IVT with rt‐PA (184, 40.6%)	rPerC (4.3 ± 0.9) Blank (6,0 ± 1.5)	c, d, e, g
Landman et al., 2022^63^ (NTR6880)	Journal of stroke	88, Netherlands	AIS	≥18	rlPostC (72.3 ± 8.9) Sham (67.4 ± 12.9)	rlPostC (28,70%) Sham (32,66.7%)	rlPostC (*n* = 40) Sham (*n* = 30)	Received IVT (50, 56.8%) and underwent Intra‐arterial thrombectomy (22, 25%)	rlPostC (9.0 ± 5.8) Sham (10.8 ± 6.4)	b, c, d, e, g
Li et al., 2018^52^ (ChiCTR‐IOR‐15006549)	Journal of Stroke and Cerebrovascular Diseases	60, China	AIS	18 ~ 75	LIPostC (68.4 ± 6.8) Sham (64.3 ± 10.0)	/	LIPostC (*n* = 29) Sham (*n* = 31)	Not received IVT	LIPostC (5.9 ± 3.0) Sham (6.1 ± 3.7)	a, b, c, f
Li et al., 2020^64^ (ChiCTR1800015231)	Journal of Stroke and Cerebrovascular Diseases	48, China	AIS	50 ~ 80	RIPC (68.3 ± 5.5) Sham (66.7 ± 6.2)	RIPC (14,58.3%) Sham (16,66.7%)	RIPC (*n* = 24) Sham (*n* = 24)	Not received IVT	RIPC (7.1 ± 2.2) Sham (7.6 ± 2.4)	a, b, f, h, j
Meng et al., 2012^38^ (NCT01321749)	Neurology	68, China	IAS with onset of AIS or TIA	18 ~ 80	BAIPC (61.1 ± 10.1) Sham (60.0 ± 9.4)	BAIPC (21,55.3%) Sham (19,63.3%)	BAIPC (*n* = 38) Sham (*n* = 30)	Not received IVT	BAIPC (11.0 ± 1.9) Sham (3.8 ± 0.7)	a, c, f, j
Meng et al., 2015^37^ (NCT01570231)	Neurotherapeutics	58, China	IAS with onset of AIS or TIA	80 ~ 95	BAIPC (83.5 ± 2.3) Sham (84.2 ± 1.6)	BAIPC (18,60.0%) Sham (17,60.7%)	BAIPC (*n* = 30) Sham (*n* = 28)	Not received IVT	BAIPC (11.4 ± 2.3) Sham (11.1 ± 2.6)	a, b, h, j
Pico et al., 2020^65^ (NCT02189928)	JAMA Neurology	188, France	AIS	≥18	rPerC (67.8 ± 15.1) Blank (66.7 ± 16.4)	rPerC (45,48.4%) Blank (53,55.8%)	rPerC (*n* = 93) Blank (*n* = 95)	Received IVT (164, 87.2%) and underwent mechanical thrombectomy (64, 34.0%)	rPerC (9.8 ± 1.8) Blank (11.0 ± 2.0)	b, c, d, e, f, g, h, i
Poalelung et al., 2021^42^ (/)	Frontiers in Neurology	40, Romania	AIS	50 ~ 80	RIC (66.8 ± 6.4) Sham (64.4 ± 9.0)	RIC (11,61.1%) Sham (13,59.1%)	RIC (*n* = 18) Sham (*n* = 22)	Not received IVT	7.7(total mean only)	a, f, g, h, i
Zhang et al., 2022^66^ (/)	Frontiers in Neurology	41, China	AIS	≥18	RIC (61.6 ± 10.9) Blank (64.3 ± 13.6)	RIC (11,57.9%) Blank (15,68.1%)	RIC (*n* = 19) Blank (*n* = 22)	Not received IVT	RIC (6.3 ± 0.9) Blank (7.0 ± 2.1)	d, f, g, h, i
Zhao et al., 2017^36^ (NCT01654666)	Circulation	189, China	after CAS with severe carotid artery stenosis	≥18	RIPC (67.5 ± 8.6) Sham (65.7 ± 8.2) Blank (66.5 ± 8.6)	RIPC (46,73.0%) Sham (44,69.8%) Blank (45,71.4%)	RIPC (*n* = 63) Sham (*n* = 63) Blank (*n* = 63)	Not received IVT	/	a, g, h, j

*Note*: a—Recurrence of ischemic stroke at the endpoint, b—NIHSS score at the endpoint, c—Excellent outcome (mRS 0–1) at 90 days, d—Favorite outcome (mRS 0–2) at 90 days, e—Dependency (mRS 3–5) at 90 days, f—RIC‐related adverse events, g—Death, h—Intracerebral hemorrhage, i—Pneumonia, j—TIA.

Abbreviations: /, no data available; AIS, acute ischemic stroke; BAIPC, bilateral arm ischemic preconditioning; Blank, only receiving standard medical management; CAS, carotid artery stenting; IAS, intracranial arterial stenosis; IS, ischemic stroke; IVT, intravenous thrombolysis; LIPostC, limb ischemic postconditioning; NIHSS, NIH Stroke Scale; RIPC, remote ischemic postconditioning; rIPostC, repeated remote ischemic postconditioning; rt‐PA, intravenous recombinant tissue plasminogen activator; TIA, transient ischemic attack.

### 3.2 Overall outcome of efficacy and safety

**FIGURE 2:**
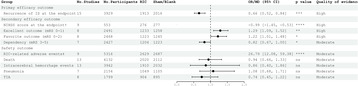
Summary of the overall efficacy and safety of RIC on ischemic stroke. ^†^NIHSS score at the endpoint was the only continuous outcome thus we did not map the forest plot in this figure. ^‡^The effect size of RIC‐related adverse events was far beyond the scale of this figure (0–2) thus we did not map the forest plot in this figure. The column of *p*‐value, statistics for testing differences between RIC and control group within one factor (ns, not significant; *p < 0.05; **p < 0.01; ***p < 0.001; ****p < 0.0001).

We pooled 6392 patients from 17 randomized controlled trials. All the forest plots exported from RevMan 5.4.1 are shown in Figure S1. RIC could reduce the recurrence of ischemic stroke at the endpoints (OR 0.66, 95% CI [0.52, 0.84]). RIC could also improve the prognosis of patients at 90 days assessed by mRS score (mRS 0–1: OR 1.29, 95% CI [1.09, 1.52]; mRS 0–2: OR 1.22, 95% CI [1.01, 1.48]) and at the endpoints assessed by NIHSS score (MD −0.99, 95% CI [−1.45, −0.53]). RIC would not cause additional adverse events such as death (*p* = 0.72), intracerebral hemorrhage events (*p* = 0.69), pneumonia (*p* = 0.75), and TIA (*p* = 0.24) but would inevitably cause RIC‐related adverse events (OR 26.79, 95% CI [12.08, 59.38]). The overall results for each outcome are shown in Figure 2. To further explore the efficacy and safety of RIC for patients diagnosed with AIS, subgroup analysis was performed and the results are summarized in Table S4.

### 3.3 Subgroup analysis for primary efficacy outcomes

RIC would reduce the recurrence of IS in the protocol of bilateral upper limbs occluded (OR 0.68, 95% CI [0.52, 0.88], *p* = 0.003), 5 cycles (OR 0.69, 95% CI [0.53, 0.89], *p* = 0.004), and 50 min (OR 0.68, 95% CI [0.52, 0.88], *p* = 0.003) of each intervention. And RIC could reduce the recurrence of IS at the endpoint only when used as the main treatment (OR 0.66, 95% CI [0.51, 0.85], *p* = 0.001) instead of as adjuvant therapy to reperfusion therapy, regardless of intervention during ischemia (*p* = 0.34) or reperfusion (*p* = 0.98).

### 3.4 Subgroup analysis for secondary efficacy outcomes

For NIHSS scores at the endpoints, RIC showed advantages over control independent of the number of limbs occluded (*p* = 0.002, *p* = 0.004) and the number of RIC cycles (*p* = 0.002, *p* = 0.004). Notably, performing RIC as main treatment instead of adjuvant to reperfusion therapy (*p* = 0.08, *p* = 0.07) still remained effective (MD −1.52, 95% CI [−2.27, −0.76], *p* < 0.0001). The subgroup analysis of excellent outcomes at 90 days and favorite outcome at 90 days showed very similar results. Not only the RIC protocol of bilateral (OR 1.30, 95% CI [1.08, 1.58], *p* = 0.007; OR 1.31, 95% CI [1.06, 1.64], *p* = 0.01), 5 cycles (OR 1.29, 95% CI [1.06, 1.55], *p* = 0.009; OR 1.31, 95% CI [1.06, 1.63], *p* = 0.01), and 50 min (OR 1.36, 95% CI [1.15, 1.60], *p* = 0.001; OR 1.27, 95% CI [1.02, 1.58], *p* = 0.04) but also initiating RIC during ischemia without reperfusion therapy (OR 1.28, 95% CI [1.05, 1.55], *p* = 0.01; OR 1.27, 95% CI [1.02, 1.58], *p* = 0.04) supported the efficacy of RIC. In terms of dependency at 90 days, RIC could improve patients' prognosis only when used on bilateral upper limbs (OR 0.77, 95% CI [0.62, 0.97], *p* = 0.02) and 5 cycles of each intervention (OR 0.77, 95% CI [0.62, 0.97], *p* = 0.02).

The results from NIHSS score at the endpoints were apparently inconsistent in the assessment of mRS. The discrepancy may be explained as follows: NIHSS scores were divided into 42 sub‐items based on neurological meticulous symptoms, whereas mRS scores were divided into 6 sub‐items based on broader indicators, suggesting that NIHSS scores are more sensitive to changes in scores induced by RIC, which might exaggerate the improvement of RIC when assessing.

### 3.5 Heterogeneity test and sensitivity analysis

We found substantial heterogeneity (*I* ^2^ = 57%, *p* = 0.02) in excellent outcomes at 90 days as a whole, subgroups of bilateral (*I* ^2^ = 86%, *p* = 0.0009), 5 cycles (*I* ^2^ = 80%, *p* = 0.002), 50 min (*I* ^2^ = 92%, *p* = 0.0004, only two studies), and no reperfusion therapy (*I* ^2^ = 84%, *p* = 0.002). After performing a Labbe plot (Figure S2), we found that Meng 2012^38^ study was responsible research for substantial heterogeneity, and the heterogeneity was reduced to below 50% after removing this study. Simultaneously, substantial heterogeneity was also observed in the subgroup of bilateral (*I* ^2^ = 55%, *p* = 0.13, only two studies) in favorite outcome at 90 days and NIHSS scores at the endpoints (*I* ^2^ = 56%, *p* = 0.13, only two studies). However, we did not test the heterogeneity for subgroups with only two studies because the presented substantial heterogeneity may be amplified results.

The heterogeneity of Meng 2012^38^ may be partly due to the different duration of intervention, of which Meng 2012 was 180 days and most studies were no more than 2 weeks (13/17, 76.5%), which reminded us that it was necessary to remove studies of chronic RIC (4/17, 23.5%) and perform a comprehensive analysis for the remaining studies (Table S5) to validate the stability of the results. Unfortunately, only chronic RIC showed efficacy in reducing the recurrence (OR 0.67, 95% CI [0.51, 0.87], *p* = 0.003) while acute (*p* = 0.27) and delayed RIC (*p* = 0.34) showed no efficacy. Inspiringly, there was no other change in the direction of the results was found.

### 3.6 Risk of bias assessment and quality of the evidence

The risk of bias summary is shown in Figure S3 and the bias of each study is shown in Figure S4. Except for one study^40^ whose randomization was based on the odevity of patient card IDs, which had a high risk of selection bias (random sequence generation), and three studies^36^, ^41^, ^42^ that did not describe the method of randomization had unclear risk, the remaining studies (13/17, 76.5%) was of low risk. The risk of bias for allocation concealment was similar to that for randomization. Notably, performance bias was significant for all the studies because blinding of receivers and operators was impossible for the intervention form of RIC, of which (7/17, 41.2%) were high risk due to non‐sham control, and (10/17, 58.8%) were unclear due to sham control. To make RCTs blinded to some degree and more convincing, blinding of outcome assessment was well performed in all included studies. Other biases, including attrition bias and reporting bias, were low in total. For publication bias, visual inspection of the contour‐enhanced funnel plots (Figure S5), and Egger's regression tests for all outcomes (*p* > 0.05) indicated low publication bias except for RIC‐related adverse events (*p* = 0.009) because they were understandably unavoidable. The results of the quality of evidence are shown in Tables S6 and S7. Except for the recurrence of ischemic stroke, excellent outcome (mRS 0–1), and favorite outcome (mRS 0–2), which were of high quality, the quality of the remaining outcomes was moderate.

## 4 DISCUSSION

This present study provides comprehensive evidence on the effects of RIC on AIS patients in RCTs. A total of 17 studies with 6392 patients were included. Based on the included studies, we found that chronic RIC could significantly reduce the recurrence of stroke at the endpoint. And RIC could improve patients' prognosis at 90 days as assessed by mRS score and at the endpoint assessed by NIHSS score. In further subgroup analysis, we found that intervention on bilateral limbs, 5 cycles, and a length of 50 min of each intervention might be an optimal protocol of RIC. In addition, RIC applied alone was effective, while there was no difference in people with AIS treated with RIC adjuvant to reperfusion therapy. Finally, RIC would not cause additional adverse events such as death, intracerebral hemorrhage events, pneumonia, and TIA but would inevitably cause benign RIC‐related adverse events.

**FIGURE 3:**
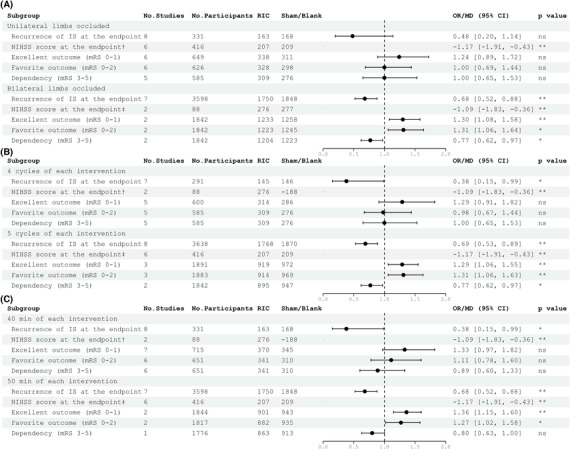
Summary of the subgroup analysis results for protocols of RIC. A. Summary of subgroup analysis results for the number of limbs occluded in all the outcomes. B. Summary of subgroup analysis results for the number of RIC cycles in all the outcomes. C. Summary of subgroup analysis results for length of each RIC intervention in all the outcomes. ^†^NIHSS score at the endpoint was the only continuous outcome thus we did not map the forest plot in this figure. ^‡^The effect size of RIC‐related adverse events was far beyond the scale of this figure (0–2) thus we did not map the forest plot in this figure. The column of *p*‐value, statistics for testing differences between RIC and control group within one factor (ns, not significant; *p < 0.05; **p < 0.01).

As shown in Figure 3A, our study showed that bilateral upper limbs applied in the RIC group could significantly improve the prognosis of patients which is different from the results of the previous preclinical meta‐analysis.^20^, ^21^ It has been reported that one in four patients with IS has silent peripheral arterial disease^43^ with a more rapid decline in lower limb‐threatening ischemia,^44^ for which it has been suggested that the upper limb would be the most appropriate to ensure safety. Most previous meta‐analyses^20^, ^21^, ^24^ showed it is effective to use both one and two limbs in RIC treatment, but they used young male rodents with a notable absence of animals with comorbidities such as age, hypertension, and diabetes factors. For these reasons, animal models would be more sensitive to RIC and subsequently amplify the effects of single limb of RIC. Another important issue is the number of cycles and the length of each RIC intervention as shown in Figure 3B,C. Consistent with previously published meta‐analyses,^21^, ^27^ studies have shown the need to increase the RIC stimulus and repetitions, which might be related to the total ischemic dose (cycle number and duration). Furthermore, in another study,^24^ there is no difference with the total length of limb occlusion (a product of cycle number and length of each cycle, reflecting the “dose” of RIC), though only doses greater than 25 min reduced infarct volume significantly, which suggested that there might be a minimum threshold value for the neuroprotective effect of RIC. Most RIC trials in IS have used the protocol of four or five cycles and 40 or 50 min, probably because preclinical studies did so.^12^, ^45^ Future studies may break through the traditional design and determine exactly how many cycles are optimal for RIC. Knowing the minimum effective number of cycles would be beneficial in the clinical setting, as we can keep the patients' exposure to RIC to a minimum duration of intervention to reduce side effects and potentially harmful sequelae.

**FIGURE 4:**
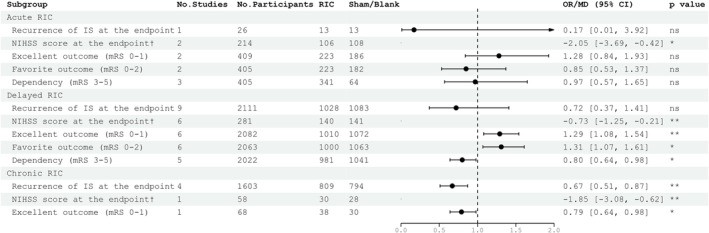
Summary of the subgroup analysis results for the type of RIC classified by duration of the intervention. ^†^NIHSS score at the endpoint was the only continuous outcome while thus we did not map the forest plot in this figure. ^‡^The effect size of RIC‐related adverse events was far beyond the scale of this figure (0–2) thus we did not map the forest plot in this figure. The column of *p*‐value, statistics for testing differences between RIC and control group within one factor (ns, not significant; *p < 0.05; **p < 0.01).

As shown in Figure 4, our analysis supported that chronic RIC might be beneficial. However, no statistical difference was found between acute and delayed RIC in reducing the recurrence of stroke. According to our studies, it is suggested that chronic RIC is a good option for patients who are not eligible for the narrow window for thrombolysis (within 4.5 h after symptom onset) and endovascular reconstruction (within 24 h after symptom onset based on two studies published online on Oct 13, 2022, in the New England Journal of Medicine,^5^, ^8^) and our result was consistent with most preclinical meta‐analyses.^20^, ^21^, ^23^, ^24^ However, one study^23^ performed within 48–72 h showed better effects compared with those performed in the hyperacute stage and 4–7 days. Given the limited evidence, the exact time window and the most effective neuroprotective RIC intervention period could not be fully determined. Further studies providing chronic RIC beyond the ischemic period should be a clinical research priority as it is important for stroke recovery. The mechanisms of chronic RIC may not only depend on reperfusion,^46^ which was important because only 50% of strokes achieve early recanalization after IV thrombolysis^27^ but also improve serum levels of cardiac enzymes and endothelial injury markers,^47^ enhance collateral circulation,^48^ improve cardiac enzymes,^49^ mediate neuroprotection through glucagon‐like‐peptide‐1 receptor activation.^50^

**FIGURE 5:**
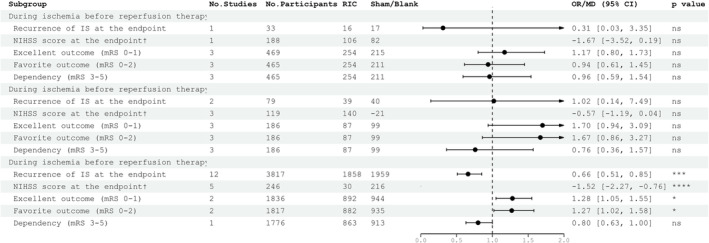
Summary of the subgroup analysis results for the time to initiate RIC. ^†^NIHSS score at the endpoint was the only continuous outcome thus we did not map the forest plot in this figure. ^‡^The effect size of RIC‐related adverse events was far beyond the scale of this figure (0–2) thus we did not map the forest plot in this figure. The column of *p*‐value, statistics for testing differences between RIC and control group within one factor (ns, not significant; *p < 0.05; ***p < 0.001; ****p < 0.0001).

As shown in Figure 5. In our study, RIC showed no significant effects in reducing the recurrence of stroke and improving prognosis in patients with acute ischemic stroke who received reperfusion therapy as assessed by NIHSS score and mRS score. However, we found that RIC (most studies were outside the treatment window for reperfusion therapy) was effective when applied alone. Inconsistent with our results, a meta‐analysis^27^ of preclinical data showed that RIC was effective during acute ischemia when used both alone and in combination with reperfusion therapies, while a meta‐analysis of the Cochrane Library^26^ (including 735 participants who underwent) supported our results. More importantly, reperfusion therapies would only be beneficial within a narrow window, beyond which the likelihood of adverse events increases.^51^ Therefore, we designed to find the optimal timing of intervention when combining RIC with IVT. Although no significant results were not obtained in patients receiving reperfusion therapy during acute ischemia or reperfusion period, several studies^38^, ^41^, ^52^, ^53^ have already demonstrated the neuroprotective effect of consecutive RIC combined with IVT during the acute ischemia period. A previous study performed RIC before IVT in patients confirmed as AIS when they were transported to the hospital and found that prehospital RIC could have immediate neuroprotective effects, demonstrating the feasibility of applying RIC before IVT.^54^ In another study, patients in the RIC group has significantly lower levels of hs‐CRP tested 24 h after thrombolysis, suggesting that RIC had an anti‐inflammatory effect in patients with AIS who underwent IVT.^53^ Consistent with our study, one meta‐analysis^20^ found no significant results during acute ischemia. A possible explanation is that the effect of intravenous thrombolysis may be so significant that it obscures the effect of RIC before and after the reperfusion period. Further RCTs will improve the current knowledge regarding RIC as an adjuvant approach in combination with reperfusion therapies for the treatment of AIS.

There were several limitations to our study. Firstly, the sample size of two studies^39^, ^55^ was significantly larger than most of other studies, which could potentially bias the results. Secondly, for those who did not receive reperfusion therapy, we did not explore the potential relationship between the time points to initiate RIC and treatment effect as in the meta‐analysis for preclinical trials because of insufficient data for subgroup analysis. Thirdly, we did not perform further subgroup analysis for trials performing acute, delayed, and chronic RIC, respectively, similarly due to insufficient data, and pooling RCTs of different RIC types together may influence clinical decisions. Fourthly, because most of the studies were conducted in China, ethnic differences might limit the extrapolation of our findings to some extent. Fifthly, in stroke intervention research, there is a heated discussion about gender differences^56^, ^57^ and some comorbidities^58^ (e.g., atherosclerosis, hypertension, diabetes, hyperlipidemia). However, these factors were present in varying proportions in both intervention and control groups in all included studies. Based on the data reported in the articles alone, we were unable to perform further subgroup or interaction analyses based on these factors in the included 6392 patients to assess their impact on the efficacy and safety of RIC. Notably, several ongoing large randomized clinical trials, ClinicalTrials.gov numbers NCT04980651 (2210 participants), NCT03481777 (1500 participants), and NCT03669653(912 participants), are expected to provide more robust evidence in future.

## 5 CONCLUSION

Overall, chronic RIC could significantly reduce the recurrence of stroke at the endpoint, and improve patients' prognosis at 90 days or the endpoints assessed by mRS score and NIHSS score. Furthermore, intervention on bilateral upper limbs, 5 cycles, and a length of 50 min in each intervention might be an optimal protocol for RIC currently. For patients ineligible for reperfusion therapy, RIC might be an effective way to recover from damage. Inspiringly, RIC might not cause other adverse events besides relatively benign RIC‐related adverse events.

## AUTHOR CONTRIBUTIONS

XK involved in drafting/revision of the manuscript for content, including medical writing for content; major role in the acquisition of data; study concept or design; and analysis or interpretation of data. ZY involved in drafting/revision of the manuscript for content, including medical writing for content; major role in the acquisition of data; and analysis or interpretation of data. FW involved in drafting/revision of the manuscript for content, including medical writing for content; major role in the acquisition of data; and analysis or interpretation of data. XT involved in drafting/revision of the manuscript for content, including medical writing for content; and analysis or interpretation of data. TX involved in drafting/revision of the manuscript for content, including medical writing for content; and analysis or interpretation of data. ZC involved in drafting/revision of the manuscript for content, including medical writing for content; and supervision and funding acquisition. ZW involved in drafting/revision of the manuscript for content, including medical writing for content; study concept or design; and supervision and funding acquisition. GC involved in drafting/revision of the manuscript for content, including medical writing for content; and project administration and funding acquisition.

## FUNDING INFORMATION

This work was supported by the Natural Science Foundation of Jiangsu Province (Grants No. BK202002023), Suzhou Health Talents Training Project (Grants No GSWS2019002), and a grant from the National Natural Science Foundation of China (No 82171294).

## CONFLICT OF INTEREST STATEMENT

The authors declare no conflicts of interest.

## Supporting information


Data S1.

## Data Availability

The data that support the findings of this study are available from the corresponding author upon reasonable request.
