# Supplementary material for: Efficacy and safety of remote ischemic conditioning for acute ischemic stroke: A comprehensive meta‐analysis from randomized controlled trials
Source: CNS Neurosci Ther. 2023 May 14;29(9):2445–56. doi: 10.1111/cns.14240 (PMC10401132; doi:10.1111/cns.14240)
Supplement: Supplementary file 1 — Data S1. [file CNS-29-2445-s001.pdf]

Supplementary Material for Review

Legends

Table S1 PRISMA 2020 Checklist

Table S2 Search strategy

Table S3 Summary of details of intervention of studies included in the meta-analysis

Table S4 Summary of the subgroup analysis results for the efficacy and safety of RIC on ischemic stroke

Table S5 Summary of the subgroup analysis results for the efficacy and safety of acute or delayed RIC on ischemic stroke

Table S6 Summary table for Quality assessment for included studies (for all studies)

Table S7 Summary table for Quality assessment for included studies (for studies performing acute or delayed RIC)

Figure S1 Forest plots for all the meta-analysis of this study exported from RevMan 5.4.1

A Forest plot of the efficacy and safety of RIC on acute ischemic stroke

B Forest plot of the efficacy and safety of acute or delayed RIC on acute ischemic stroke

Figure S2 Heterogeneity test of excellent outcome (mRS 0-1) at 90 days

A Labbe plot for all studies (I2 = 57%, 0% after removing Meng 2012)

B Labbe plot for subgroup of studies using bilateral upper limbs (I2 = 86%, 42% after removing Meng 2012)

C Labbe plot for subgroup of studies using 5 cycles of each intervention (I2 = 80%, 18% after removing Meng 2012)

D Labbe plot for subgroup of studies without reperfusion therapy (I2 = 84%, 0% after removing Meng 2012)

Figure S3 Summary table for potential bias analysis for included studies

Figure S4 Summary table for potential bias analysis for individual studies

Figure S5 Contour-enhanced funnel plots for all the outcomes (p value of Egger's test)

Table S1 PRISMA 2020 Checklist

Section and Topic	Item #	Checklist item	Location where item is reported
**TITLE**			
Title	1	Identify the report as a systematic review.	1
**ABSTRACT**			
Abstract	2	See the PRISMA 2020 for Abstracts checklist.	1
**INTRODUCTION**			
Rationale	3	Describe the rationale for the review in the context of existing knowledge.	2
Objectives	4	Provide an explicit statement of the objective(s) or question(s) the review addresses.	3
**METHODS**			
Eligibility criteria	5	Specify the inclusion and exclusion criteria for the review and how studies were grouped for the syntheses.	4
Information sources	6	Specify all databases, registers, websites, organisations, reference lists and other sources searched or consulted to identify studies. Specify the date when each source was last searched or consulted.	4
Search strategy	7	Present the full search strategies for all databases, registers and websites, including any filters and limits used.	4, Table S2
Selection process	8	Specify the methods used to decide whether a study met the inclusion criteria of the review, including how many reviewers screened each record and each report retrieved, whether they worked independently, and if applicable, details of automation tools used in the process.	4
Data collection process	9	Specify the methods used to collect data from reports, including how many reviewers collected data from each report, whether they worked independently, any processes for obtaining or confirming data from study investigators, and if applicable, details of automation tools used in the process.	5

Section and Topic	Item #	Checklist item	Location where item is reported
Data items	10a	List and define all outcomes for which data were sought. Specify whether all results that were compatible with each outcome domain in each study were sought (e.g. for all measures, time points, analyses), and if not, the methods used to decide which results to collect.	5
	10b	List and define all other variables for which data were sought (e.g. participant and intervention characteristics, funding sources). Describe any assumptions made about any missing or unclear information.	5
Study risk of bias assessment	11	Specify the methods used to assess risk of bias in the included studies, including details of the tool(s) used, how many reviewers assessed each study and whether they worked independently, and if applicable, details of automation tools used in the process.	6
Effect measures	12	Specify for each outcome the effect measure(s) (e.g. risk ratio, mean difference) used in the synthesis or presentation of results.	5-6
Synthesis methods	13a	Describe the processes used to decide which studies were eligible for each synthesis (e.g. tabulating the study intervention characteristics and comparing against the planned groups for each synthesis (item #5)).	Table 1 and Table S3
	13b	Describe any methods required to prepare the data for presentation or synthesis, such as handling of missing summary statistics, or data conversions.	5
	13c	Describe any methods used to tabulate or visually display results of individual studies and syntheses.	5
	13d	Describe any methods used to synthesize results and provide a rationale for the choice(s). If meta-analysis was performed, describe the model(s), method(s) to identify the presence and extent of statistical heterogeneity, and software package(s) used.	5
	13e	Describe any methods used to explore possible causes of heterogeneity among study results (e.g. subgroup analysis, meta-regression).	6, 10 and Figure S1
	13f	Describe any sensitivity analyses conducted to assess robustness of the synthesized results.	6
Reporting	14	Describe any methods used to assess risk of bias due to missing results in a synthesis (arising	/

Section and Topic	Item #	Checklist item	Location where item is reported
bias assessment		from reporting biases).	
Certainty assessment	15	Describe any methods used to assess certainty (or confidence) in the body of evidence for an outcome.	7
**RESULTS**			
Study selection	16a	Describe the results of the search and selection process, from the number of records identified in the search to the number of studies included in the review, ideally using a flow diagram.	8, Figure 1
	16b	Cite studies that might appear to meet the inclusion criteria, but which were excluded, and explain why they were excluded.	Figure 1
Study characteristics	17	Cite each included study and present its characteristics.	Table 1
Risk of bias in studies	18	Present assessments of risk of bias for each included study.	10, Figure S3, Figure S4
Results of individual studies	19	For all outcomes, present, for each study: (a) summary statistics for each group (where appropriate) and (b) an effect estimate and its precision (e.g. confidence/credible interval), ideally using structured tables or plots.	Figure 2, Figure 3-5
Results of syntheses	20a	For each synthesis, briefly summarise the characteristics and risk of bias among contributing studies.	10
	20b	Present results of all statistical syntheses conducted. If meta-analysis was done, present for each the summary estimate and its precision (e.g. confidence/credible interval) and measures of statistical heterogeneity. If comparing groups, describe the direction of the effect.	Table S4 and Figure S2.A
	20c	Present results of all investigations of possible causes of heterogeneity among study results.	10
	20d	Present results of all sensitivity analyses conducted to assess the robustness of the synthesized results.	10, Table S5 and

Section and Topic	Item #	Checklist item	Location where item is reported
			Figure S2.B
Reporting biases	21	Present assessments of risk of bias due to missing results (arising from reporting biases) for each synthesis assessed.	/
Certainty of evidence	22	Present assessments of certainty (or confidence) in the body of evidence for each outcome assessed.	Table S6 and Table S7
**DISCUSSION**			
Discussion	23a	Provide a general interpretation of the results in the context of other evidence.	11
	23b	Discuss any limitations of the evidence included in the review.	13
	23c	Discuss any limitations of the review processes used.	13
	23d	Discuss implications of the results for practice, policy, and future research.	13-14
**OTHER INFORMATION**			
Registration and protocol	24a	Provide registration information for the review, including register name and registration number, or state that the review was not registered.	4
	24b	Indicate where the review protocol can be accessed, or state that a protocol was not prepared.	4
	24c	Describe and explain any amendments to information provided at registration or in the protocol.	/
Support	25	Describe sources of financial or non-financial support for the review, and the role of the funders or sponsors in the review.	14
Competing interests	26	Declare any competing interests of review authors.	14
Availability of data, code and other materials	27	Report which of the following are publicly available and where they can be found: template data collection forms; data extracted from included studies; data used for all analyses; analytic code; any other materials used in the review.	14

From: Page MJ, McKenzie JE, Bossuyt PM, Boutron I, Hoffmann TC, Mulrow CD, et al. The PRISMA 2020 statement: an updated guideline for reporting systematic reviews. BMJ 2021;372:n71. doi: 10.1136/bmj.n71

For more information, visit: http://www.prisma-statement.org/

Table S2 Search strategy

**1. Pubmed**	
# 1	Search: (((((((remote ischemic conditioning [Title/Abstract]) OR (RIC[Title/Abstract])) OR (remote ischemic preconditioning [Title/Abstract])) OR (RIPreC[Title/Abstract])) OR (remote ischemic perconditioning[Title/Abstract])) OR (RIPerC[Title/Abstract])) OR (remote ischemic postconditioning[Title/Abstract])) OR (RIPostC[Title/Abstract]))
# 2	Search: ((((((((((((((((((((((((Strokes[Title/Abstract]) OR (Cerebrovascular Accident[Title/Abstract])) OR (Cerebrovascular Accidents[Title/Abstract])) OR (CVAs (Cerebrovascular Accident[Title/Abstract]))) OR (Cerebrovascular Apoplexy[Title/Abstract])) OR (Apoplexy, Cerebrovascular[Title/Abstract])) OR (Vascular Accident, Brain[Title/Abstract])) OR (Brain Vascular Accident[Title/Abstract])) OR (Brain Vascular Accidents[Title/Abstract])) OR (Vascular Accidents, Brain[Title/Abstract])) OR (Cerebrovascular Stroke[Title/Abstract])) OR (Cerebrovascular Strokes[Title/Abstract])) OR (Stroke, Cerebrovascular[Title/Abstract])) OR (Strokes, Cerebrovascular[Title/Abstract])) OR (Apoplexy[Title/Abstract])) OR (Cerebral Stroke[Title/Abstract])) OR (Cerebral Strokes[Title/Abstract])) OR (Stroke, Cerebral[Title/Abstract])) OR (Strokes, Cerebral[Title/Abstract])) OR (Stroke, Acute[Title/Abstract])) OR (Acute Stroke[Title/Abstract])) OR (Acute Strokes[Title/Abstract])) OR (Strokes, Acute[Title/Abstract])) OR (Cerebrovascular Accident, Acute[Title/Abstract])) OR (Acute Cerebrovascular Accident[Title/Abstract])) OR (Acute Cerebrovascular Accidents[Title/Abstract])) OR (Cerebrovascular Accidents, Acute[Title/Abstract])) OR ("Stroke"[Mesh]))
# 3	Search: (#1) AND (#2) 136
**2. Embase**	
# 1	((RIC) OR (remote ischemic conditioning) OR (RlPreC) OR (remote ischemic preconditioning) OR (RlPerC) OR (remote ischemic perconditioning) OR (RlPostC) OR (remote ischemic postconditioning))

# 2	(Stroke OR strokes OR (Cerebrovascular Accident) OR (Cerebrovascular Accidents) OR CVA (Cerebrovascular Accident) OR CVAs (Cerebrovascular Accident) OR (Cerebrovascular Apoplexy) OR (Apoplexy, Cerebrovascular) OR (Vascular Accident, Brain) OR (Brain Vascular Accident) OR (Brain Vascular Accidents) OR (Vascular Accidents, Brain) OR (Cerebrovascular Stroke) OR (Cerebrovascular Strokes) OR (Stroke, Cerebrovascular) OR (Strokes, Cerebrovascular) OR Apoplexy OR (Cerebral Stroke) OR (Cerebral Strokes) OR (Stroke, Cerebral) OR (Strokes, Cerebral) OR (Stroke, Acute) OR (Acute Stroke) OR (Acute Strokes) OR (Strokes, Acute) OR (Cerebrovascular Accident, Acute) OR (Acute Cerebrovascular Accident) OR (Acute Cerebrovascular Accidents) OR (Cerebrovascular Accidents, Acute))
# 3	#1 AND #2 319

3. Cochrane Library

# 1	(((((((remote ischemic conditioning) OR (RIC)) OR (remote ischemic preconditioning)) OR (RIPreC)) OR (remote ischemic perconditioning)) OR (RIPerC)) OR (remote ischemic postconditioning)) OR (RIPostC))
# 2	((((((((((((((((((((((((((Strokes) OR (Cerebrovascular Accident)) OR (Cerebrovascular Accidents)) OR (CVAs (Cerebrovascular Accident))) OR (Cerebrovascular Apoplexy)) OR (Apoplexy, Cerebrovascular)) OR (Vascular Accident, Brain)) OR (Brain Vascular Accident)) OR (Brain Vascular Accidents)) OR (Vascular Accidents, Brain)) OR (Cerebrovascular Stroke)) OR (Cerebrovascular Strokes)) OR (Stroke, Cerebrovascular)) OR (Strokes, Cerebrovascular)) OR (Apoplexy)) OR (Cerebral Stroke)) OR (Cerebral Strokes)) OR (Stroke, Cerebral)) OR (Strokes, Cerebral)) OR (Stroke, Acute)) OR (Acute Stroke)) OR (Acute Strokes)) OR (Strokes, Acute)) OR (Cerebrovascular Accident, Acute)) OR (Acute Cerebrovascular Accident)) OR (Acute Cerebrovascular Accidents)) OR (Cerebrovascular Accidents, Acute)) OR ("Stroke"))
# 3	(# 1) AND (# 2) 215

4. ClinicalTrials.gov

# 1	(Remote Ischemic Conditioning) OR RIC OR (remote ischemic preconditioning) OR RIPreC OR (remote ischemic perconditioning) OR RIPerC OR (remote ischemic postconditioning) OR RIPostC | stroke OR (Cerebrovascular Accident) OR (Cerebrovascular Stroke) OR (Apoplexy) OR (Cerebral Stroke) OR (Stroke, Cerebral) OR (Acute Stroke) OR (Acute Cerebrovascular Accident) 58

5. Elsevier

# 1	("Remote Ischemic Conditioning" OR "RIC" OR "remote ischemic preconditioning" OR "RIPreC" OR "remote ischemic perconditioning" OR "RIPerC" OR "remote ischemic postconditioning" OR "RIPostC") AND ("stroke" OR "Cerebrovascular Accident" OR "Cerebrovascular Stroke" OR "Apoplexy" OR "Cerebral Stroke" OR "Stroke, Cerebral" OR "Acute Stroke" OR "Acute Cerebrovascular Accident") 571

Table S3 Summary of details of intervention of studies included in the meta-analysis

Study	Type of RIC tested	Number of limbs occluded	Number of RIC cycles	Length of each RIC intervention	Total length of limb ischemia	Time to initiate RIC
An 2020 (NCT03218293)	RIPC (b)	unilateral upper arm (the transfusion side was avoided)	5	40 min	6 d	within 3.5 h of IVT (administered intravenous t-PA within 4.5 h of stroke onset)
Che 2019 (NCT03231384)	RIPC (b)	bilateral upper limbs	5	40 min	7 d	within 2 h of IVT (administered intravenous rt-PA within 4.5 h of stroke onset)
Chen 2022 (NCT03740971)	RIC (b)	bilateral upper limbs	5	50 min	10~14 d	within 48 h after stroke onset (within 24.8±13.2 h in RIC group or 25.0 ± 13.7 h in control group)
England 2017 (ISRCTN86672015)	RIC (a)	unilateral upper arm (the nonparetic arm)	4	40 min	＜= 24 h	within 24 h after stroke onset
England 2019 (NCT02779712)	RIC (b)	unilateral upper arm (the nonparetic arm)	4	40 min	4 d	within 6 h after stroke onset

He 2020 (NCT04027621)	RIC (b)	unilateral upper arm (the nonparetic arm)	4	40 min	24 h	within 24 h of IVT (administered intravenous rt-PA within 4.5 h of stroke onset)
Hou 2022 (NCT02534545)	RIC (c)	bilateral upper limbs	5	45 min	1 y	Within 30 days of stroke onset or transient ischaemic attack up to 15 days
Hougaard 2013 (NCT00975962)	rPerC (a)	unilateral upper arm (the nonparetic arm)	4	40 min	＜= 24 h	during ambulance transportation
Landman 2022 (NTR6880)	rlPostC (b)	unilateral upper arm (the nonparetic arm)	4	40 min	＜4 h	within 11.8 ± 5.9 h in rlPostC group or 14.1 ± 6.6 h in control group after stroke onset
Li 2018 (ChiCTR-IOR15006549)	LIPostC (b)	unilateral upper arm (the nonparetic arm)	4	40 min	14 d	within 72 h after stroke onset
Li 2020 (ChiCTR1800015231)	RIPC (b)	unilateral upper arm (the nonparetic arm)	4	40 min	7 d	within 72 h after stroke onset
Meng 2012 (NCT01321749)	BAIPC (c)	bilateral upper limbs	5	50 min	300 d	within 30 d after stroke or TIA onset

Meng 2015 (NCT01570231)	BAIPC (c)	bilateral upper limbs	5	50 min	180 d	within 7 d after stroke or TIA onset
Pico 2020 (NCT02189928)	rPerC (a)	unilateral upper arm (the nonparetic arm)	4	40 min	24 h	within 6 h after stroke onset (with an average of 3.7 h)
Poalelung 2021 (/)	RIC (b)	unilateral upper arm (the nonparetic arm)	5	40 min	5 d	within 24 d after stroke onset
Zhang 2022 (/)	RIC (b)	unilateral upper arm (the transfusion side was avoided)	5	50 min	6 d	Randomization was performed within 48 h of onset and started within 2 hours of onset
Zhao 2017 (NCT01654666)	RIPC (/)	bilateral upper limbs	5	50 min	14 d	within 6 months after symptomatic carotid artery stenosis

a. Recurrence of ischemic stroke with acute RIC, b. Recurrence of ischemic stroke with delayed RIC, c. Recurrence of ischemic stroke with chronic RIC, /: we did not include Zhao 2017 in any of three types and corresponding analysis because participants was diagnosed with internal carotid artery stenosis while only treated for 2 weeks.

Table S4 Summary of the subgroup analysis results for the efficacy and safety of RIC on ischemic stroke

Outcome or subgroup title	No. of studies	No. of participants	Effect size	*p* value
1 Primary outcome				
1.1 Recurrence of ischemic stroke at the endpoint	15	3929	**0.66 [0.52, 0.84] *** **	**0.0009*** **
1.1.1 Number of limbs occluded				
Unilateral	8	331	0.48 [0.20, 1.14]	0.10
Bilateral	7	3598	**0.68 [0.52, 0.88] ** **	**0.003 ** **
1.1.2 Number of RIC cycles				
4 cycles	7	291	0.38 [0.15, 0.99]	0.05
5 cycles	8	3638	**0.69 [0.53, 0.89] ** **	**0.004 ** **
1.1.3 Length of each RIC intervention				
40 min	8	331	0.48 [0.20, 1.14]	0.10
50 min	7	3598	**0.68 [0.52, 0.88] ** **	**0.003 ** **
1.1.4 Type of RIC				
Acute RIC	1	26	0.17 [0.01, 3.92]	0.27

Delayed RIC	9	2111	0.72 [0.37, 1.41]	0.34
Chronic RIC	4	1603	**0.67 [0.51, 0.87] ****	**0.003 ****
1.1.5 Time to initiation of RIC				
During ischemia before reperfusion therapy	1	33	0.31 [0.03, 3.35]	0.34
During reperfusion after reperfusion therapy	2	79	1.02 [0.14, 7.49]	0.98
During ischemia without reperfusion therapy	12	3817	**0.66 [0.51, 0.85] ****	**0.001****
2 Secondary outcome				
2.1 NIHSS score at the endpoint †	9	553	**-0.99 [-1.45, -0.53] *****	**< 0.0001*****
2.1.1 Number of limbs occluded				
Unilateral	6	416	**-1.17 [-1.91, -0.43] ****	**0.002****
Bilateral ‡	2	88	**-1.09 [-1.83, -0.36] ****	**0.004****
2.1.2 Number of RIC cycles				
4 cycles	6	416	**-1.17 [-1.91, -0.43] ****	**0.002****

5 cycles ǂ	2	88	**-1.09 [-1.83, -0.36] ****	**0.004****
2.1.3 Length of each RIC intervention				
40 min	6	416	**-1.17 [-1.91, -0.43] ****	**0.002****
50 min ǂ	2	88	**-1.09 [-1.83, -0.36] ****	**0.004****
2.1.4 Type of RIC				
Acute RIC	2	214	**-2.05 [-3.69, -0.42] ***	**0.01***
Delayed RIC	6	281	**-0.73 [-1.25, -0.21] ****	**0.006****
Chronic RIC	1	58	**-1.85 [-3.08, -0.62] ****	**0.003****
2.1.5 Time to initiate the RIC				
During ischemia before reperfusion therapy	1	188	-1.67 [-3.52, 0.19]	0.08
During reperfusion after reperfusion therapy	3	119	-0.57 [-1.19, 0.04]	0.07
During ischemia without reperfusion therapy	5	246	**-1.52 [-2.27, -0.76] *****	**< 0.0001*****

2.2 Excellent outcome (mRS 0-1) at 90days	8	2491	**1.29 [1.09, 1.52] ** **	**0.003** **
2.2.1 Number of limbs occluded				
Unilateral	6	649	1.24 [0.89, 1.72]	0.20
Bilateral	2	1842	**1.30 [1.08, 1.58] ** **	**0.007** **
2.2.2 Number of RIC cycles				
4 cycles	5	600	1.29 [0.91, 1.82]	0.15
5 cycles	3	1891	**1.29 [1.06, 1.55] ** **	**0.009** **
2.2.3 Length of each RIC intervention				
40 min	7	715	1.33 [0.97, 1.82]	0.07
50 min ‡	2	1844	**1.36 [1.15, 1.60] ** **	**0.001** **
2.2.4 Type of RIC				
Acute RIC	2	409	1.28 [0.84, 1.93]	0.25
Delayed RIC	6	2082	**1.29 [1.08, 1.54] ** **	**0.006 ** **
Chronic RIC	1	68	**12.50 [3.59, 43.54] *** ***	**< 0.0001*** **
2.2.5 Time to initiate the RIC				

During ischemia before reperfusion therapy	3	469	1.17 [0.80, 1.73]	0.42
During reperfusion after reperfusion therapy	3	186	1.70 [0.94, 3.09]	0.08
During ischemia without reperfusion therapy	2	1836	**1.28 [1.05, 1.55] ***	**0.01***
2.3 Favourite outcome (mRS 0-2) at 90days	8	2468	**1.22 [1.01, 1.48] ***	**0.04***
2.3.1 Number of limbs occluded				
Unilateral	6	626	1.00 [0.69, 1.44]	0.99
Bilateral ‡	2	1842	**1.31 [1.06, 1.64] ***	**0.01***
2.3.2 Number of RIC cycles				
4 cycles	5	585	0.98 [0.67, 1.44]	0.93
5 cycles	3	1883	**1.31 [1.06, 1.63] ***	**0.01***
2.3.3 Length of each RIC intervention				
40 min	6	651	1.11 [0.78, 1.60]	0.55
50 min	2	1817	**1.27 [1.02, 1.58] ***	**0.04***
2.3.4 Type of RIC				
Acute RIC	2	405	0.85 [0.53, 1.37]	0.52
Delayed RIC	6	2063	**1.31 [1.07, 1.61] ***	**0.01***

2.3.5 Time to initiate the RIC				
During ischemia before reperfusion therapy	3	465	0.94 [0.61, 1.45]	0.78
During reperfusion after reperfusion therapy	3	186	1.67 [0.86, 3.27]	0.13
During ischemia without reperfusion therapy	2	1817	**1.27 [1.02, 1.58] ***	**0.04***
2.4 Dependency (mRS 3-5) at 90 days	7	2427	0.82 [0.67, 1.00]	0.05
2.4.1 Number of limbs occluded				
Unilateral	5	585	1.00 [0.65, 1.53]	1.00
Bilateral	2	1842	**0.77 [0.62, 0.97] ***	**0.02***
2.4.2 Number of RIC cycles				
4 cycles	5	585	1.00 [0.65, 1.53]	1.00
5 cycles	2	1842	**0.77 [0.62, 0.97] ***	**0.02***
2.4.3 Length of each RIC intervention				
40 min	6	651	0.89 [0.60, 1.33]	0.57
50 min	1	1776	0.80 [0.63, 1.00]	0.05
2.4.4 Type of RIC				
Acute RIC	2	405	0.97 [0.57, 1.65]	0.91
Delayed RIC	5	2022	**0.80 [0.64, 0.98] ***	**0.04 ***

2.4.5 Time to initiate the RIC				
During ischemia before reperfusion therapy	3	465	0.96 [0.59, 1.54]	0.86
During reperfusion after reperfusion therapy	3	186	0.76 [0.36, 1.57]	0.45
During ischemia without reperfusion therapy	1	1776	0.80 [0.63, 1.00]	0.05
3 Safety				
3.1 RIC-related adverse events	9	5316	**26.79 [12.08, 59.38] *** **	**< 0.00001*** **
3.2 Death	13	4132	0.94 [0.66, 1.33]	0.72
3.2.1 Number of limbs occluded				
Unilateral	9	692	1.16 [0.63, 2.12]	0.63
Bilateral	4	3440	0.84 [0.55, 1.29]	0.44
3.2.2 Number of RIC cycles				
4 cycles	7	611	1.10 [0.59, 2.05]	0.77
5 cycles	6	3521	0.87 [0.58, 1.33]	0.53
3.2.3 Length of each RIC intervention				
40 min	9	717	1.06 [0.59, 1.88]	0.85
50 min	4	3415	0.88 [0.57, 1.35]	0.55

3.2.4 Type of RIC				
Acute RIC	3	431	1.73 [0.78, 3.84]	0.17
Delayed RIC	8	2103	0.63 [0.33, 1.23]	0.18
Chronic RIC	1	1409	0.92 [0.56, 1.49]	0.72
3.2.5 Time to initiate the RIC				
During ischemia before reperfusion therapy	3	438	1.42 [0.68, 2.95]	0.35
During reperfusion after reperfusion therapy	3	186	0.41 [0.11, 1.44]	0.16
During ischemia without reperfusion therapy	7	3508	0.91 [0.59, 1.38]	0.65
3.3 Intracerebral hemorrhagic events	13	3942	0.86 [0.40, 1.86]	0.69
3.3.1 Number of limbs occluded				
Unilateral	7	414	0.71 [0.28, 1.81]	0.47
Bilateral	6	3528	1.32 [0.32, 5.40]	0.70
3.3.2 Number of RIC cycles				
4 cycles	5	333	0.71 [0.28, 1.81]	0.47
5 cycles	8	3745	1.15 [0.42, 3.12]	0.79
3.3.3 Length of each RIC intervention				
40 min	7	439	0.74 [0.30, 1.79]	0.50

50 min	6	3503	1.41 [0.28, 7.25]	0.68
3.3.4 Type of RIC				
Acute RIC	1	176	1.00 [0.24, 4.13]	1.00
Delayed RIC	9	2110	0.75 [0.26, 2.15]	0.60
Chronic RIC	2	1467	1.00 [0.14, 7.15]	1.00
3.3.5 Time to initiate the RIC				
During ischemia before reperfusion therapy	2	209	1.25 [0.35, 4.46]	0.73
During reperfusion after reperfusion therapy	3	145	0.64 [0.16, 2.55]	0.53
During ischemia without reperfusion therapy	8	3588	0.73 [0.18, 3.02]	0.66
3.4 Pheumonia	7	2154	1.08 [0.68, 1.71]	0.75
3.4.1 Number of limbs occluded				
Unilateral	6	378	0.67 [0.31, 1.42]	0.29
Bilateral	1	1776	1.46 [0.80, 2.66]	0.21
3.4.2 Number of RIC cycles				
4 cycles	4	297	0.82 [0.35, 1.91]	0.64
5 cycles ǂ	3	1857	1.21 [0.70, 2.11]	0.50
3.4.3 Length of each RIC intervention				

40 min	5	337	0.82 [0.35, 1.91]	0.64
50 min ‡	2	1817	1.21 [0.70, 2.11]	0.50
3.4.4 Type of RIC				
Acute RIC	1	188	0.73 [0.24, 2.20]	0.58
Delayed RIC	6	1966	1.17 [0.70, 1.96]	0.54
3.4.5 Time to initiate the RIC				
During ischemia before reperfusion therapy	2	221	0.55 [0.20, 1.48]	0.24
During reperfusion after reperfusion therapy	1	49	3.26 [0.13, 83.90]	0.48
During ischemia without reperfusion therapy	4	1884	1.28 [0.75, 2.20]	0.37
3.5 TIA	8	1799	0.74 [0.45, 1.22]	0.24
3.5.1 Number of limbs occluded				
Unilateral	3	108	2.85 [0.28, 28.73]	0.37
Bilateral	5	1691	0.68 [0.41, 1.14]	0.15
3.5.2 Number of RIC cycles				
4 cycles	3	108	2.85 [0.28, 28.73]	0.37
5 cycles	5	1691	0.68 [0.41, 1.14]	0.15
3.5.3 Length of each RIC intervention				

40 min	3	108	2.85 [0.28, 28.73]	0.37
50 min	5	1691	0.68 [0.41, 1.14]	0.15
3.5.4 Type of RIC				
Delayed RIC	4	138	1.66 [0.37, 7.51]	0.51
Chronic RIC	3	1535	0.66 [0.38, 1.13]	0.13
3.5.5 Time to initiate the RIC				
During ischemia before reperfusion therapy	1	33	Not estimable	Not applicable
During reperfusion after reperfusion therapy	1	30	1.00 [0.12, 8.21]	1.00
During ischemia without reperfusion therapy	6	1736	0.73 [0.44, 1.21]	0.22

[†]. Continuous outcome, Mean Difference (IV, Fixed, 95% CI); the remaining outcomes were dichotomous outcomes, Odds Ratio (M-H, Fixed, 95% CI)

*p < 0.05; **p < 0.01; ***p < 0.001 and bold. Statistically significant results: OR 95% CI, MD 95% CI, *p* value

[‡]. Substantial heterogeneity (I^2 > 50%) which may be amplified results because there were only two studies in corresponding subgroups. So the presented effect value might be not credible.

Table S5: Summary of the subgroup analysis results for the efficacy and safety of acute or delayed RIC on ischemic stroke

Outcome or subgroup title	No. of studies	No. of participants	Effect size	*p* value
1 Primary outcome				
1.1 Recurrence of ischemic stroke at the endpoint	10	2137	0.66 [0.35, 1.27]	0.21
1.1.1 Number of limbs occluded				
Unilateral	8	331	0.48 [0.20, 1.14]	0.10
Bilateral	2	1806	1.05 [0.38, 2.91]	0.92
1.1.2 Number of RIC cycles				
4 cycles	7	291	0.41 [0.17, 1.00]	0.05
5 cycles	3	1846	1.20 [0.47, 3.07]	0.70
1.1.3 Length of each RIC intervention				
40 min	8	331	0.48 [0.20, 1.14]	0.10
50 min	2	1806	1.05 [0.38, 2.91]	0.92
1.1.4 Type of RIC				
Acute RIC	1	26	0.17 [0.01, 3.92]	0.27
Delayed RIC	9	2111	0.72 [0.37, 1.41]	0.34
1.1.5 Time to initiation of RIC				

During ischemia before reperfusion therapy	1	33	0.31 [0.03, 3.35]	0.34
During reperfusion after reperfusion therapy	2	79	1.02 [0.14, 7.49]	0.98
During ischemia without reperfusion therapy	7	2025	0.68 [0.33, 1.40]	0.29
2 Secondary outcome				
2.1 NIHSS score at the endpoint †	8	495	**-0.85 [-1.35, -0.36] *** **	**< 0.0001*** **
2.1.1 Number of limbs occluded				
Unilateral	7	465	**-0.93 [-1.52, -0.34] ** **	**0.002** **
Bilateral ‡	1	30	-0.67 [-1.59, 0.26]	0.16
2.1.2 Number of RIC cycles				
4 cycles	7	465	**-0.93 [-1.52, -0.34] ** **	**0.002** **
5 cycles ‡	1	30	-0.67 [-1.59, 0.26]	0.16
2.1.3 Length of each RIC intervention				
40 min	7	465	**-0.93 [-1.52, -0.34] ** **	**0.002** **
50 min ‡	1	30	-0.67 [-1.59, 0.26]	0.16

2.1.4 Type of RIC				
Acute RIC	2	214	**-2.05 [-3.69, -0.42] ***	**0.01***
Delayed RIC	6	281	**-0.73 [-1.25, -0.21] ****	**0.006****
2.1.5 Time to initiate the RIC				
During ischemia before reperfusion therapy	1	188	-1.67 [-3.52, 0.19]	0.08
During reperfusion after reperfusion therapy	3	119	-0.57 [-1.19, 0.04]	0.07
During ischemia without reperfusion therapy	4	188	**-1.31 [-2.27, -0.36] ****	**0.007****
2.2 Excellent outcome (mRS 0-1) at 90days	8	2491	**1.29 [1.09, 1.52] ****	**0.003****
2.2.1 Number of limbs occluded				
Unilateral	6	649	1.24 [0.89, 1.72]	0.20
Bilateral	2	1842	**1.30 [1.08, 1.58] ****	**0.007****
2.2.2 Number of RIC cycles				
4 cycles	5	600	1.29 [0.91, 1.82]	0.15
5 cycles	3	1891	**1.29 [1.06, 1.55] ****	**0.009****

2.2.3 Length of each RIC intervention				
40 min	7	715	1.33 [0.97, 1.82]	0.07
50 min	1	1776	**1.27 [1.04, 1.54] ***	**0.02***
2.2.4 Type of RIC				
Acute RIC	2	409	1.28 [0.84, 1.93]	0.25
Delayed RIC	6	2082	**1.29 [1.08, 1.54] ****	**0.006 ****
2.2.5 Time to initiate the RIC				
During ischemia before reperfusion therapy	3	469	1.17 [0.80, 1.73]	0.42
During reperfusion after reperfusion therapy	3	186	1.70 [0.94, 3.09]	0.08
During ischemia without reperfusion therapy	2	1836	**1.28 [1.05, 1.55] ***	**0.01***
2.3 Favourite outcome (mRS 0-2) at 90days	8	2468	**1.22 [1.01, 1.48] ***	**0.04***
2.3.1 Number of limbs occluded				
Unilateral	6	626	1.00 [0.69, 1.44]	0.99
Bilateral ‡	2	1842	**1.31 [1.06, 1.64] ***	**0.01***
2.3.2 Number of RIC cycles				

4 cycles	5	585	0.98 [0.67, 1.44]	0.93
5 cycles	3	1883	**1.31 [1.06, 1.63] ***	**0.01***
2.3.3 Length of each RIC intervention				
40 min	6	651	1.11 [0.78, 1.60]	0.55
50 min	2	1817	**1.27 [1.02, 1.58] ***	**0.04***
2.3.4 Type of RIC				
Acute RIC	2	405	0.85 [0.53, 1.37]	0.52
Delayed RIC	6	2063	**1.31 [1.07, 1.61] ***	**0.01***
2.3.5 Time to initiate the RIC				
During ischemia before reperfusion therapy	3	465	0.94 [0.61, 1.45]	0.78
During reperfusion after reperfusion therapy	3	186	1.67 [0.86, 3.27]	0.13
During ischemia without reperfusion therapy	2	1817	**1.27 [1.02, 1.58] ***	**0.04***
2.4 Dependency (mRS 3-5) at 90 days	7	2427	0.82 [0.67, 1.00]	0.05
2.4.1 Number of limbs occluded				
Unilateral	5	585	1.00 [0.65, 1.53]	1.00
Bilateral	2	1842	**0.77 [0.62, 0.97] ***	**0.02***
2.4.2 Number of RIC cycles				

4 cycles	5	585	1.00 [0.65, 1.53]	1.00
5 cycles	2	1842	**0.77 [0.62, 0.97] ***	**0.02***
2.4.3 Length of each RIC intervention				
40 min	6	651	0.89 [0.60, 1.33]	0.57
50 min	1	1776	0.80 [0.63, 1.00]	0.05
2.4.4 Type of RIC				
Acute RIC	2	405	0.97 [0.57, 1.65]	0.91
Delayed RIC	5	2022	**0.80 [0.64, 0.98] ***	**0.04***
2.4.5 Time to initiate the RIC				
During ischemia before reperfusion therapy	3	465	0.96 [0.59, 1.54]	0.86
During reperfusion after reperfusion therapy	3	186	0.76 [0.36, 1.57]	0.45
During ischemia without reperfusion therapy	1	1776	0.80 [0.63, 1.00]	0.05
3 Safety				
3.1 RIC-related adverse events	7	2184	**10.76 [3.24, 35.79] *** **	**0.0001*** **
3.2 Death	11	2534	0.96 [0.59, 1.57]	0.87
3.2.1 Number of limbs occluded				

Unilateral	9	692	1.16 [0.63, 2.12]	0.63
Bilateral	2	1842	0.65 [0.27, 1.57]	0.34
3.2.2 Number of RIC cycles				
4 cycles	7	611	1.10 [0.59, 2.05]	0.77
5 cycles	4	1923	0.77 [0.34, 1.74]	0.52
3.2.3 Length of each RIC intervention				
40 min	9	717	1.06 [0.59, 1.88]	0.85
50 min	2	1817	0.74 [0.28, 1.95]	0.54
3.2.4 Type of RIC				
Acute RIC	3	431	1.73 [0.78, 3.84]	0.17
Delayed RIC	8	2103	0.63 [0.33, 1.23]	0.18
3.2.5 Time to initiate the RIC				
During ischemia before reperfusion therapy	3	438	1.42 [0.68, 2.95]	0.35
During reperfusion after reperfusion therapy	3	186	0.41 [0.11, 1.44]	0.16
During ischemia without reperfusion therapy	5	1910	0.87 [0.37, 2.04]	0.76
3.3 Intracerebral hemorrhagic events	10	2286	0.83 [0.36, 1.93]	0.67
3.3.1 Number of limbs occluded				

Unilateral	7	414	0.71 [0.28, 1.81]	0.47
Bilateral	3	1872	1.76 [0.22, 13.91]	0.59
3.3.2 Number of RIC cycles				
4 cycles	5	333	0.71 [0.28, 1.81]	0.47
5 cycles	5	2089	1.20 [0.38, 3.84]	0.79
3.3.3 Length of each RIC intervention				
40 min	7	439	0.74 [0.30, 1.79]	0.50
50 min	3	1847	3.21 [0.12, 85.20]	0.49
3.3.4 Type of RIC				
Acute RIC	1	176	1.00 [0.24, 4.13]	1.00
Delayed RIC	9	2110	0.75 [0.26, 2.15]	0.60
3.3.5 Time to initiate the RIC				
During ischemia before reperfusion therapy	2	209	1.25 [0.35, 4.46]	0.73
During reperfusion after reperfusion therapy	3	145	0.64 [0.16, 2.55]	0.53
During ischemia without reperfusion therapy	5	1932	0.51 [0.06, 4.18]	0.53
3.4 Pheumonia	7	2154	1.08 [0.68, 1.71]	0.75
3.4.1 Number of limbs occluded				

Unilateral	6	378	0.67 [0.31, 1.42]	0.29
Bilateral	1	1776	1.46 [0.80, 2.66]	0.21
3.4.2 Number of RIC cycles				
4 cycles	4	297	0.82 [0.35, 1.91]	0.64
5 cycles ‡	3	1857	1.21 [0.70, 2.11]	0.50
3.4.3 Length of each RIC intervention				
40 min	5	337	0.82 [0.35, 1.91]	0.64
50 min ‡	2	1817	1.21 [0.70, 2.11]	0.50
3.4.4 Type of RIC				
Acute RIC	1	188	0.73 [0.24, 2.20]	0.58
Delayed RIC	6	1966	1.17 [0.70, 1.96]	0.54
3.4.5 Time to initiate the RIC				
During ischemia before reperfusion therapy	2	221	0.55 [0.20, 1.48]	0.24
During reperfusion after reperfusion therapy	1	49	3.26 [0.13, 83.90]	0.48
During ischemia without reperfusion therapy	4	1884	1.28 [0.75, 2.20]	0.37
3.5 TIA	4	138	1.66 [0.37, 7.51]	0.51
3.5.1 Number of limbs occluded				

Unilateral	3	108	2.85 [0.28, 28.73]	0.37
Bilateral	1	30	1.00 [0.12, 8.21]	1.00
3.5.2 Number of RIC cycles				
4 cycles	3	108	2.85 [0.28, 28.73]	0.37
5 cycles	1	30	1.00 [0.12, 8.21]	1.00
3.5.3 Length of each RIC intervention				
40 min	3	108	2.85 [0.28, 28.73]	0.37
50 min	1	30	1.00 [0.12, 8.21]	1.00
3.5.4 Type of RIC				
Delayed RIC	4	138	1.66 [0.37, 7.51]	0.51
3.5.5 Time to initiate the RIC				
During ischemia before reperfusion therapy	1	33	Not estimable	Not applicable
During reperfusion after reperfusion therapy	1	30	1.00 [0.12, 8.21]	1.00
During ischemia without reperfusion therapy	2	75	2.85 [0.28, 28.73]	0.37

[†]. Continuous outcome, Mean Difference (IV, Fixed, 95% CI); the remaining outcomes were dichotomous outcomes, Odds Ratio (M-H, Fixed, 95% CI)

*p < 0.05; **p < 0.01; ***p < 0.001 and bold. Statistically significant results: OR 95% CI, MD 95% CI, *p* value

‡. Substantial heterogeneity (I^2 > 50%) which may be amplified results because there were only two studies in corresponding subgroups. So the presented effect value might be not credible.

Table S6 Summary table for Quality assessment for included studies (for all studies)

RIC versus non-RIC for treating acute ischemic stroke

Patient or population: patients with acute ischemic stroke

Settings: emergency, clinic and inpatient

Intervention: RIC

Outcomes	Illustrative comparative risks* (95% CI)		Relative effect (95% CI)	No of Participants (studies)	Quality of the evidence (GRADE)	Comments
	Assumed risk	Corresponding risk				
	**Control**	**RIC**				
**recurrence of ischemic stroke at the endpoint**	**Study population**		**OR 0.66** (0.52 to 0.84)	3929 (15 studies)	⊕⊕⊕⊕ **high**[1]	
	**88 per 1000**	**60 per 1000** (48 to 75)				
	**Moderate**					
	**83 per 1000**	**56 per 1000** (45 to 71)				
**NIHSS score at the endpoint** Follow-up: mean 3		The mean NIHSS score at the endpoint in the intervention groups was **0.99 lower** (1.45 to 0.53 lower)		553 (9 studies)	⊕⊕⊕⊕ **high**	
**excellent outcome (mRS 0-1) at 90 days**	**Study population**		**OR 1.29** (1.09 to 1.52)	2491 (8 studies)	⊕⊕⊕⊕ **high**	
	**587 per 1000**	**647 per 1000** (607 to 683)				
	**Moderate**					
	**475 per 1000**	**539 per 1000** (497 to 579)				
	**Study population**					

**favourite outcome (mRS 0-2) at 90 days**	**744 per 1000**<br>**Moderate**	**780 per 1000**<br>(746 to 811)	**OR 1.22**<br>(1.01 to 1.48)	2468<br>(8 studies)	⊕⊕⊕⊕<br>**high**
	**705 per 1000**	**745 per 1000**<br>(707 to 780)			
**dependency (mRS 3-5) at 90 days**	**Study population**		**OR 0.82**<br>(0.67 to 1)	2427<br>(7 studies)	⊕⊕⊕⊖<br>**moderate**[1]
	**226 per 1000**	**193 per 1000**<br>(163 to 226)			
	**Moderate**				
	**231 per 1000**	**198 per 1000**<br>(168 to 231)			
**RIC-related adverse events**	**Study population**		**OR 26.79**<br>(12.08 to 59.38)	5316<br>(9 studies)	⊕⊕⊕⊖<br>**moderate**[1]
	**1 per 1000**	**29 per 1000**<br>(13 to 62)			
	**Moderate**				
	**0 per 1000**	**0 per 1000**<br>(0 to 0)			
**death**	**Study population**		**OR 0.94**<br>(0.66 to 1.33)	4132<br>(13 studies)	⊕⊕⊕⊖<br>**moderate**[1]
	**34 per 1000**	**32 per 1000**<br>(22 to 44)			
	**Moderate**				
	**46 per 1000**	**43 per 1000**<br>(31 to 60)			
**intracerebral hemorrhage**	**Study population**		**OR 0.86**<br>(0.4 to 1.86)	3942<br>(13 studies)	⊕⊕⊕⊖<br>**moderate**[1]
	**6 per 1000**	**6 per 1000**<br>(3 to 12)			
	**Moderate**				
	**0 per 1000**	**0 per 1000**<br>(0 to 0)			

**Pneumonia**	**Study population**	**OR 1.08** (0.68 to 1.71)	2154 (7 studies)	⊕⊕⊕⊖ **moderate**[1]
	**33 per 1000**	**35 per 1000** (22 to 54)		
	**Moderate**			
	**21 per 1000**	**23 per 1000** (14 to 35)		
**TIA**	**Study population**	**OR 0.74** (0.45 to 1.22)	1799 (8 studies)	⊕⊕⊕⊖ **moderate**[1]
	**42 per 1000**	**32 per 1000** (20 to 51)		
	**Moderate**			
	**23 per 1000**	**17 per 1000** (10 to 28)		

*The basis for the **assumed risk** (e.g. the median control group risk across studies) is provided in footnotes. The **corresponding risk** (and its 95% confidence interval) is based on the assumed risk in the comparison group and the **relative effect** of the intervention (and its 95% CI).

CI: Confidence interval; **OR:** Odds ratio;

GRADE Working Group grades of evidence

High quality: Further research is very unlikely to change our confidence in the estimate of effect.

Moderate quality: Further research is likely to have an important impact on our confidence in the estimate of effect and may change the estimate.

Low quality: Further research is very likely to have an important impact on our confidence in the estimate of effect and is likely to change the estimate.

Very low quality: We are very uncertain about the estimate.

[1] No explanation was provided

Table S7 Summary table for Quality assessment for included studies (for studies performing acute or delayed RIC)

acute or delayed RIC versus non-RIC for treating acute ischemic stroke

Patient or population: patients with acute ischemic stroke

Settings: emergency, clinic and inpatient

Intervention: acute or delayed RIC

Outcomes	Illustrative comparative risks* (95% CI)		Relative effect (95% CI)	No of Participants (studies)	Quality of the evidence (GRADE)	Comments
	Assumed risk<br>**Control**	Corresponding risk<br>**Acute or delayed RIC**				
**recurrence of ischemic stroke at the endpoint**	**Study population**		**OR 0.66** (0.35 to 1.27)	2137 (10 studies)	⊕⊕⊕⊕ **high**	
	**19 per 1000**	**13 per 1000** (7 to 24)				
	**Moderate**					
	**75 per 1000**	**51 per 1000** (28 to 93)				
**NIHSS score at the endpoint**		The mean NIHSS score at the endpoint in the intervention groups was **0.85 lower** (1.35 to 0.36 lower)		495 (8 studies)	⊕⊕⊕⊕ **high**	
**excellent outcome (mRS 0-1) at 90 days**	**Study population**		**OR 1.29** (1.09 to 1.52)	2491 (8 studies)	⊕⊕⊕⊕ **high**	
	**587 per 1000**	**647 per 1000** (607 to 683)				
	**Moderate**					
	**488 per 1000**	**551 per 1000** (510 to 592)				
	**Study population**					

**favourite outcome (mRS 0-2) at 90 days**	**744 per 1000**<br>**Moderate**	**780 per 1000**<br>(746 to 811)	**OR 1.22**<br>(1.01 to 1.48)	2468<br>(8 studies)	⊕⊕⊕⊕<br>**high**
	**705 per 1000**	**745 per 1000**<br>(707 to 780)			
**dependency (mRS 3-5) at 90 days**	**Study population**		**OR 0.82**<br>(0.67 to 1)	2427<br>(7 studies)	⊕⊕⊕⊖<br>**moderate**[1]
	**226 per 1000**	**193 per 1000**<br>(163 to 226)			
	**Moderate**				
	**231 per 1000**	**198 per 1000**<br>(168 to 231)			
**RIC-related adverse events**	**Study population**		**OR 10.76**<br>(3.24 to 35.79)	2184<br>(7 studies)	⊕⊕⊕⊖<br>**moderate**[1]
	**0 per 1000**	**0 per 1000**<br>(0 to 0)			
	**Moderate**				
	**0 per 1000**	**0 per 1000**<br>(0 to 0)			
**death**	**Study population**		**OR 0.96**<br>(0.59 to 1.57)	2534<br>(11 studies)	⊕⊕⊕⊖<br>**moderate**[1]
	**27 per 1000**	**26 per 1000**<br>(16 to 42)			
	**Moderate**				
	**46 per 1000**	**44 per 1000**<br>(28 to 70)			
**intracerebral hemorrhage**	**Study population**		**OR 0.83**<br>(0.36 to 1.93)	2286<br>(10 studies)	⊕⊕⊕⊖<br>**moderate**[1]
	**9 per 1000**	**8 per 1000**<br>(3 to 18)			
	**Moderate**				
	**15 per 1000**	**12 per 1000**<br>(5 to 29)			

**Pneumonia**	**Study population**	**OR 1.08** (0.68 to 1.71)	2154 (7 studies)	⊕⊕⊕⊖ **moderate**[1]
	**33 per 1000**	**35 per 1000** (22 to 54)		
	**Moderate**			
	**21 per 1000**	**23 per 1000** (14 to 35)		
**TIA**	**Study population**	**OR 1.66** (0.37 to 7.51)	138 (4 studies)	⊕⊕⊕⊖ **moderate**[1]
	**29 per 1000**	**48 per 1000** (11 to 185)		
	**Moderate**			
	**0 per 1000**	**0 per 1000** (0 to 0)		

*The basis for the **assumed risk** (e.g. the median control group risk across studies) is provided in footnotes. The **corresponding risk** (and its 95% confidence interval) is based on the assumed risk in the comparison group and the **relative effect** of the intervention (and its 95% CI).

CI: Confidence interval; **OR:** Odds ratio;

GRADE Working Group grades of evidence

High quality: Further research is very unlikely to change our confidence in the estimate of effect.

Moderate quality: Further research is likely to have an important impact on our confidence in the estimate of effect and may change the estimate.

Low quality: Further research is very likely to have an important impact on our confidence in the estimate of effect and is likely to change the estimate.

Very low quality: We are very uncertain about the estimate.

[1] No explanation was provided
